# Comprehensive Analysis of the Immune-Oncology Targets and Immune Infiltrates of *N*^6^-Methyladenosine-Related Long Noncoding RNA Regulators in Breast Cancer

**DOI:** 10.3389/fcell.2021.686675

**Published:** 2021-07-02

**Authors:** Xiaoqiang Zhang, Li Shen, Ruyu Cai, Xiafei Yu, Junzhe Yang, Xian Wu, Yanhui Zhu, Xiaoan Liu

**Affiliations:** Department of Breast Surgery, The First Affiliated Hospital of Nanjing Medical University, Nanjing, China

**Keywords:** breast cancer, *N*^6^-methyladenosine, immune-oncology targets, immune infiltrates, prognosis

## Abstract

Breast cancer (BRCA) has become the highest incidence of cancer due to its heterogeneity. To predict the prognosis of BRCA patients, sensitive biomarkers deserve intensive investigation. Herein, we explored the role of *N*^6^-methyladenosine-related long non-coding RNAs (m^6^A-related lncRNAs) as prognostic biomarkers in BRCA patients acquired from The Cancer Genome Atlas (TCGA; *n* = 1,089) dataset and RNA sequencing (RNA-seq) data (*n* = 196). Pearson’s correlation analysis, and univariate and multivariate Cox regression were performed to select m^6^A-related lncRNAs associated with prognosis. Twelve lncRNAs were identified to construct an m^6^A-related lncRNA prognostic signature (m^6^A-LPS) in TCGA training (*n* = 545) and validation (*n* = 544) cohorts. Based on the 12 lncRNAs, risk scores were calculated. Then, patients were classified into low- and high-risk groups according to the median value of risk scores. Distinct immune cell infiltration was observed between the two groups. Patients with low-risk score had higher immune score and upregulated expressions of four immune-oncology targets (CTLA4, PDCD1, CD274, and CD19) than patients with high-risk score. On the contrary, the high-risk group was more correlated with overall gene mutations, Wnt/β-catenin signaling, and JAK-STAT signaling pathways. In addition, the stratification analysis verified the ability of m^6^A-LPS to predict prognosis. Moreover, a nomogram (based on risk score, age, gender, stage, PAM50, T, M, and N stage) was established to evaluate the overall survival (OS) of BRCA patients. Thus, m^6^A-LPS could serve as a sensitive biomarker in predicting the prognosis of BRCA patients and could exert positive influence in personalized immunotherapy.

## Introduction

Breast cancer (BRCA) has become the highest incidence of human cancer, accounting for 11.7% of global new cancer cases in 2020, according to the latest data provided by the International Agency for Research on Cancer (IARC) (Ferlay et al., 2020). Treatments for BRCA have progressed in recent years, including chemotherapy, surgery, targeted therapies, hormone replacement therapy, radiation therapy, complementary therapies, gene therapy, and stem cell therapy (Harbeck and Gnant, 2017). Conventionally, tumor size, nodal status, hormonal receptor status, and the existence of metastatic are employed to evaluate the therapeutic strategies and survival outcomes. However, the traditional diagnostic methods cannot satisfy the advanced diagnosis and treatments. Besides, the heterogeneity of BRCA is significant, leading to the diversity of tumor evolution scenarios, thus limiting the application of conventional methods. Hence, it is urgent to identify sensitive biomarkers for accurate prognostic prediction of patients with BRCA and help improve personalized therapy managements.

*N*^6^-methyladenosine (m^6^A) is one of the most common and abundant modifications among more than 100 post-transcriptional modifications found in RNA species. It plays a major important role in biological processes including stem cell biology, tumor development, immunology, and cancer biology (Huang et al., 2020; Wang et al., 2020). The level of m^6^A is tightly regulated by methyltransferases (METTL3, METTL14, and WTAP), m^6^A-interacting proteins (YTHDF2 and YTHDF3), and demethylases (FTO and ALKBH5) (Hong, 2018). Recently, solid evidence suggests that aberrant regulation of m^6^A is in connection with various kinds of human cancers, including BRCA. Zhang et al. (2019) reported that m^6^A-loss-mediated activation of oncogenic signaling (such as Wnt and PI3K-Akt) could promote the progression of gastric cancer. As presented in Chen et al.’s (2019) research, m^6^A RNA methylation regulators, including WTAP, YTHDC1, and FTO, could be used for a prognostic prediction of bladder cancer. YTHDF1 was found to be involved in promoting ovarian cancer progression *via* controlling EIF3C’s translation in Liu et al.’s (2020b) investigation. Niu et al. (2019) explored the role of FTO in the promotion of BRCA progression through downregulation of BNIP3. Long non-coding RNAs (lncRNAs; >200 nucleotides) are significant in the pathogenesis of cancers (Qi et al., 2016). Several studies prove the association among lncRNAs and the progression of specific subtypes of BRCA. In Yi et al.’s (2019) research, lnc-SLC4A1-1 was activated by H3K27ac acetylation, promoting the development of BRCA. And the downregulated expressions of lnc-ANGPTL1-3:3 and lnc-GJA10-12:1 are important regulators of sentinel lymph node (SLN) metastasis in BRCA (Sun et al., 2020a). However, few efforts have been devoted to the research of the role of m^6^A regulators in the dysregulation of lncRNAs in BRCA. Therefore, with the aid of genome sequencing technology and bioinformatics, the investigation of m^6^A-related lncRNAs focuses on the potential biomarkers in the survival outcomes of BRCA.

In this work, based on The Cancer Genome Atlas (TCGA) dataset and RNA sequencing (RNA-seq) data derived from our previous work, the prognostic value of m^6^A-related lncRNAs could be obtained by bioinformatics and statistical analysis. Then, 12 lncRNAs with strong correlations with prognosis were filtrated and employed to construct the m^6^A-related lncRNAs prognostic signature (m^6^A-LPS). The risk scores of the BRCA patients could be derived from the m^6^A-LPS. Then, the patients with BRCA could be classified into two groups (the high- and low-risk groups) according to the median risk scores. Considering effects of immune mechanism, the variations of the risk scores were further explored. And the tumor hallmarks were more common in the high-risk group. Moreover, the nomogram model was designed to evaluate the prognosis of BRCA patients with different clinical characteristics. Effective guidance could be offered by m^6^A-LPS to predict the survival outcome for BRCA.

## Results

### Identification of m^6^A-Related Long Non-coding RNAs in Breast Cancer Patients

**FIGURE 1:**
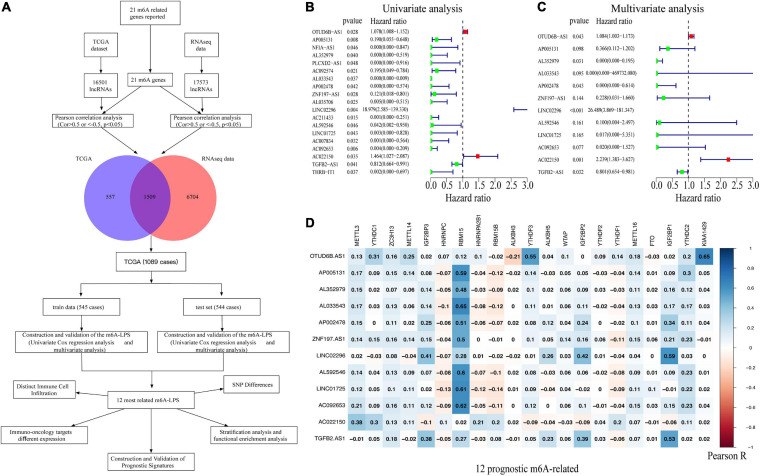
**(A)** Study flowchart. **(B,C)** Forest plots of m^6^A-related lncRNAs associated with prognosis *via* univariate and multivariate analyses. **(D)** Heatmap of the correlations between m^6^A-related genes and the 12 prognostic m^6^A-related lncRNAs. m^6^A-related lncRNAs, *N*^6^-methyladenosine-related long non-coding RNAs.

In our study, 16,501 lncRNAs from TCGA dataset and 17,573 lncRNAs (196 patients diagnosed with BRCA) from the RNA-seq data were identified. An m^6^A-related lncRNA was defined by a lncRNA whose expression was associated with one or more of the 21 m^6^A-related genes reported (| Pearson R| > 0.5 and *p* < 0.05). Figure 1A illustrates the study flowchart of this work. According to Pearson’s correlation analysis, 1,509 m^6^A-related lncRNAs in both two datasets were filtrated. Then, 1,089 BRCA patient samples obtained from TCGA dataset were randomly divided into the training cohort (545 cases) and validation cohort (544 cases). With the employment of univariate and multivariate Cox regression analysis, 12 of the 1,509 m^6^A-related lncRNAs were linked to the overall survival (OS) of BRCA patients (Figures 1B,C). Among them, OTUD6B-AS1, LINC02296, and AC022150 were defined as risk factors with hazard ratio (HR) values >1, whereas the remaining nine lncRNAs, TGFB2-AS1, LINC01725, AP002478, AL352979, AL033543, ZNF197-AS1, AL592546, AC092653, and AP005131, were defined as protective factors with HR values <1 (Figure 1C). Additionally, the correlations between the m^6^A-related genes and the 12 prognostic m^6^A-related lncRNAs were also displayed (Figure 1D).

### Construction and Validation of the m^6^A-LPS in the Cancer Genome Atlas Cohort

**FIGURE 2:**
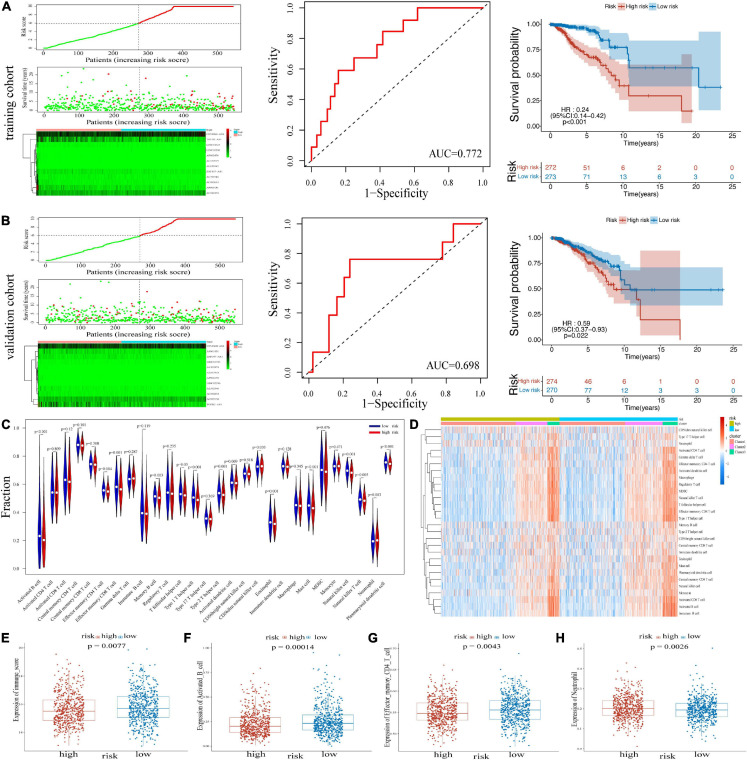
Heatmap, distribution of risk scores, survival status, and Kaplan–Meier curves of OS for BRCA patients in TCGA training cohort **(A)** and validation cohort **(B)**. **(C)** The infiltrating levels of 22 immune cell types in high-/low-risk subtypes. *p < 0.05 and **p < 0.01. **(D)** Heatmap of correlations between 22 immune cell types with risk scores and clusters. **(E)** Immune score in the high- and low-risk groups. **(F–H)** Activated B cell, effector memory CD4 T cell, and neutrophil in two risk groups. OS, overall survival; BRCA, breast cancer; TCGA, The Cancer Genome Atlas.

An m^6^A-LPS consisting of the 12 m^6^A-related lncRNAs was constructed. Based on the different expressions of 12 lncRNAs, risk scores were calculated. The distribution of risk scores and survival time of patients in the training cohort and validation cohort was respectively shown in Figures 2A,B. The heatmap results demonstrated that the expression of protective lncRNAs (including TGFB2-AS1, LINC01725, AP002478, AL352979, AL033543, ZNF197-AS1, AL592546, AC092653, and AP005131) increased with decreasing risk score, while risky lncRNAs (OTUD6B-AS1, LINC02296, and AC022150) were upregulated with increasing risk score. Besides, in the training cohort, the area under the receiver operating characteristic (ROC) curve (AUC) value for the risk signatures was 0.772; and in the validation cohort, the AUC value was 0.698. Moreover, BRCA patients in the training cohort and the validation cohort were both classified into two groups (high- and low-risk groups) by median risk score. The Kaplan–Meier curves in Figures 2A,B revealed that patients with low-risk scores had better OS than patients with high-risk scores in the two cohorts. Thereby, the ability of the risk score based on 12 risk signatures in predicting the prognosis of BRCA patients was proved.

### Association of m^6^A-LPS With Distinct Immune Cell Infiltration and Immune-Oncology Targets

**FIGURE 3:**
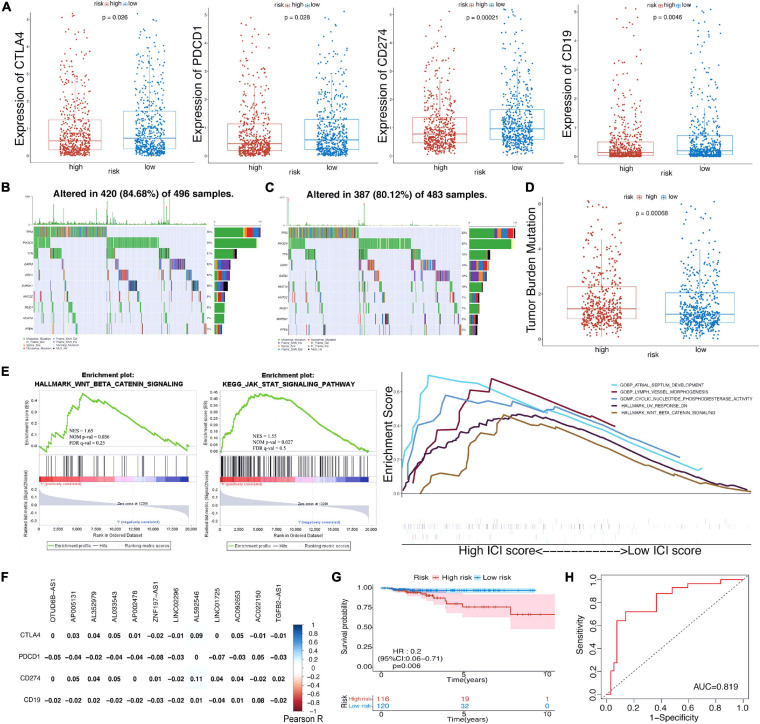
**(A)** The expression level of CTLA4, PDCD1, CD274, and CD19 in high-/low-risk subtypes in TCGA cohort. **(B,C)** Waterfall maps of eight mutated genes in high-/low-risk subtypes. **(D)** The difference of tumor mutational burden between the high- and low-risk subtypes. **(E)** Gene set enrichment analysis (GSEA) showed the tumor hallmarks enriched in the high-risk subgroup and the relevance between biological process, molecular function, and hallmarks and m^6^A-LPS. **(F)** Heatmap of the correlations between four immune targets and the 12 prognostic m^6^A-related lncRNAs. **(G)** Kaplan–Meier curves of OS for BRCA patients in the chemotherapy subgroup. **(H)** ROC curve for patients with chemotherapy. TCGA, The Cancer Genome Atlas; OS, overall survival; BRCA, breast cancer; ROC, receiver operating characteristic; lncRNAs, long non-coding RNAs.

We investigated the immune infiltrate levels between the high- and low-risk groups for exploring the interactions of m^6^A-LPS with tumor immune microenvironment (TIME) of BRCA (Figure 2C). Between the two groups, the fraction of 28 immune cell types was analyzed. Obviously, the infiltration levels of CD56dim natural killer cell, neutrophil, were higher in the high-risk group, while the low-risk group was more associated with activated B cell, effector memory CD4 T cell, effector memory CD8 T cell, memory B cell, type 1 T helper cell, type 2 T helper cell, eosinophil, mast cell, natural killer cell, natural killer T cell, and plasmacytoid dendritic cell. Moreover, the association of 28 immune cell types with different risk scores and clusters is shown in Figure 2D. The difference of immune score in two groups was equally significant, and the low-risk group had higher immune score (Figure 2E). And the results revealed that the infiltration levels of activated B cell and effector memory CD4 T cell were lower in the high-risk group in Figures 2F,G. Figure 2H displays the higher infiltration level of neutrophil in the high-risk group. In addition, the differences of the remaining immune cell types between the two groups are shown in Supplementary Figures 1A–J. To assess the correlation of immune-oncology targets with m^6^A-LPS, we compared their different expressions in risk models including two subtypes. It illustrated that the expressions of CTLA4, PDCD1, CD274, and CD19 were all distinctly unregulated in the low-risk group and lower in the high-risk group (Figure 3A). And the correlations of the four targets with 12 lncRNAs were also evaluated (Figure 3F).

### Risk Scores Correlated With Single-Nucleotide Polymorphisms, Tumor Mutation Burden, Gene Set Enrichment Analysis, and Chemotherapy

As revealed in Figures 3B,C, the condition of single-nucleotide polymorphisms (SNPs) in the risk model was further analyzed. Among genes altered in 420 (84.68%) of the 496 samples with high-risk scores obtained from TCGA dataset, eight genes were proved to have higher expression than others. TP53, PIK3CA, and TTN account for 36, 34, and 17%, respectively (Figure 3B). In the low-risk group, the expressions of eight genes including TP53 (33%), PIK3CA (32%), and CDH1 (15%) were higher than those of others altered in 387 (80.12%) of 483 samples (Figure 3C). And patients with high-risk scores had a significantly higher tumor mutational burden than patients with low-risk scores (Figure 3D). Besides, gene set enrichment analysis (GSEA) suggested that the high-risk group was more associated with Wnt/β-catenin signaling and JAK-STAT signaling pathways (Figure 3E). Cyclic nucleotide phosphodiesterase activity [normalized enrichment score (NES) = 1.86, nominal (NOM) *p*-value = 0.002, false discovery rate (FDR) *q*-value = 0.36] was the most relevant molecular function of the m^6^A-LPS; and Wnt/beta-catenin signaling pathway (NES = 1.65, NOM *p*-value = 0.036, FDR *q*-value = 0.25) was the most relevant cancer hallmark.

Furthermore, we explored the prognostic value of risk score for BRCA patients with chemotherapy. Obviously, patients with the application of chemotherapy had better survival outcome whether in the high-risk group or low-risk group (Supplementary Figures 2A,–B). Then, the subgroup (patients with chemotherapy) was further analyzed. Supplementary Figure 2C displayed the distribution of risk scores and survival time of the subgroup. It was observed that patients with high-risk scores had worse survival outcome in the subgroup (Figure 3G), and the AUC value was 0.819 (Figure 3H). The AUC values for patients with 3. 5-, 5-, and 7.5-year survival times were 0.748, 0.825, and 0.738, respectively (Supplementary Figure 2D). To further understand the effects of the risk scores on drug response, we assessed the association between risk scores and the IC_50_ of nelarabine, ZM-336372, cyclophosphamide, and dexamethasone Decadron from CellMiner database. For lack of sufficient data, only ZNF197-AS1 of the 12 lncRNAs was identified. Significant differences of IC_50_ could not be discovered between the high and low expressions of ZNF197-AS1 groups (Supplementary Figure 3A). A significant positive correlation was observed between the expression of ZNF197-AS1 and IC_50_ of Nelarabine (*p* < 0.001), ZM-336372 (*p* = 0.002), cyclophosphamide (*p* = 0.007), and dexamethasone Decadron (*p* = 0.007; Supplementary Figure 3B).

### Stratification Analysis and Independent Prognostic Value of m^6^A-LPS

**FIGURE 4:**
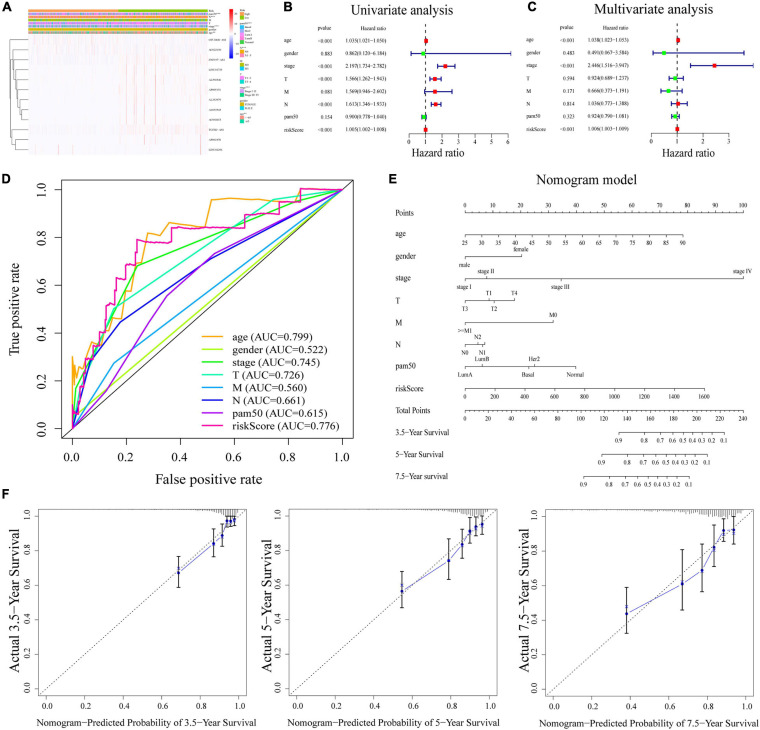
**(A)** Heatmap of the association between the expression levels of the 12 m^6^A-related lncRNAs and clinicopathological features in The Cancer Genome Atlas (TCGA) dataset. **(B,C)** Risk score was an independent prognostic predictor by univariate and multivariate analyses. **(D)** ROC curves for the risk score, age, gender, stage, T, M, N, and PAM50. **(E)** Nomogram based on risk score, age, gender, stage, T, M, N, and PAM50. **(F)** Calibration plots of the nomogram for predicting the probability of OS at 3.5, 5, and 7.5 years. ROC, receiver operating characteristic; lncRNAs, long non-coding RNAs.

The heatmap (Figure 4A) demonstrated that TGFB2-AS1, LINC01725, AP002478, AL352979, AL033543, ZNF197-AS1, AL592546, AC092653, and AP005131 expressions decreased with increasing risk score, whereas the expressions of the OTUD6B-AS1, LINC02296, and AC022150 increased with increasing risk score. Their expression levels were also related to the clinicopathological features of BRCA, such as age, gender, stage, T, M, N, and PAM50. Results in the research suggested that clinicopathological features (including age, gender, stage, T stage, M stage, N stage, and PAM50 intrinsic subtypes) were linked to the risk scores. The Kaplan–Meier curves showed that patients with the following features (aged ≤65, female, stage I–II, T1–2, M0, N0, N1–3, Basal, Her2, and LumA subtypes) all had better OS with low-risk scores (Supplementary Figure 4).

Next, univariate and multivariate Cox regression analyses were applied to prove that m^6^A-LPS was an independent prognostic factor for BRCA patients. Univariate Cox regression analysis illustrated that the risk score based on m^6^A-related lncRNAs was significantly associated with OS (HR: 1.005, 95% CI: 1.002–1.008, *p* < 0.001; Figure 4B). Furthermore, multivariate Cox regression analyses indicated that m^6^A-LPS was able to independently predict the prognosis for BRCA (HR: 1.006, 95% CI: 1.003–1.009, *p* < 0.001; Figure 4C). The ROC curve in Figure 4D shows that the AUC value for the m^6^A-LPS was 0.776, which was higher than the AUC values for gender (AUC = 0.522), stage (AUC = 0.745), T stage (AUC = 0.726), M stage (AUC = 0.560), N stage (AUC = 0.661), and PAM50 (AUC = 0.615).

### Construction of the m^6^A-LPS-Based Nomogram

A nomogram based on m^6^A-LPS was established to estimate the 3. 5-, 5-, and 7.5-year survival by using risk score and other clinicopathological factors such as age, gender, stage, PAM50, T, M, and N stage (Figure 4E). Herein, as showed in Figure 4F, the actual 3. 5-, 5-, and 7.5-year survival times were consistent with the predicted ones by calibration plots of the nomogram.

### External Validation of the m^6^A-LPS in the RNA-Sequencing Data and Comparison With the Signature Including Protein-Coding Genes

**FIGURE 5:**
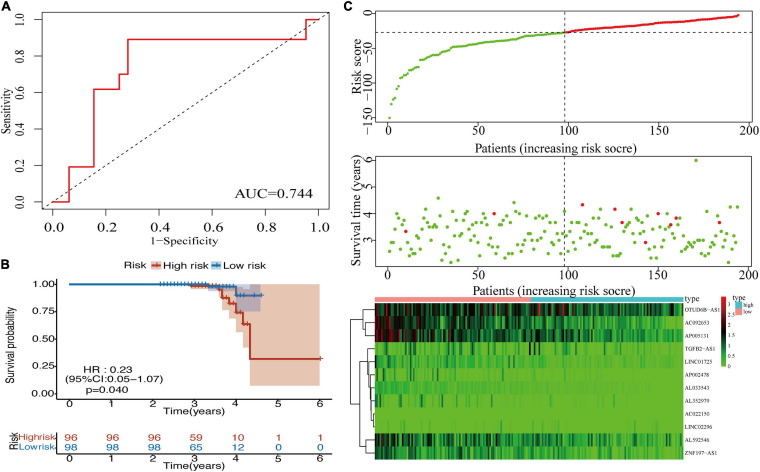
**(A)** ROC curve for the 12 lncRNAs in the external validation cohort. **(B)** Kaplan–Meier curve of OS for BRCA patients in the external validation cohort. **(C)** Heatmap, distribution of risk scores, and survival status in the external validation cohort. ROC, receiver operating characteristic; lncRNAs, long non-coding RNAs; OS, overall survival; BRCA, breast cancer.

To validate the prognostic value of the m^6^A-LPS in BRCA, an external validation cohort was designed, consisting of 196 cases from our RNA-seq data. The AUC value (0.744) for the risk signatures is shown in Figure 5A. Patients in the low-risk group had better OS than patients in the high-risk group (Figure 5B). Additionally, Figure 5C displays the distribution of risk scores and survival time of patients in the external validation cohort. The association of 12 lncRNAs with risk scores was also observed in the heatmap (Figure 5C). These findings were consistent with the analysis of TCGA data. Furthermore, we added the protein-coding genes to the signature. The Kaplan–Meier curve was shown, and the AUC value for the risk signatures including protein-coding genes was 0.677, which was lower than that of m^6^A-related lncRNA signature (AUC = 0.749; Supplementary Figure 5). Considering this, m^6^A-related lncRNA signature was constructed to predict the prognosis of BRCA patients.

## Discussion

Breast cancer is one of the most frequent causes of cancer death for females globally. Although recent advances in early diagnosis and effective treatment diminish mortality, many patients still succumb to metastasis due to therapeutic limitation and disease recurrence. The extreme heterogeneity of BRCA in histology and molecular brings certain differences in incidence, biology, treatment sensitiveness, and prognosis (Holm et al., 2017; Yeo and Guan, 2017). TIME consisting of endothelial cells, fibroblast, macrophages, and a variety of other infiltrating immune cells plays a critical role in tumor growth and metastasis (Brown et al., 2020). Tumor heterogeneity and its interaction with immune cells in the tumor microenvironment lead to the challenge for BRCA immunotherapy.

*N*^6^-methyladenosine methylation, discovered in the 1970s, is the abundant internal modification of mRNA and lncRNA in the majority of eukaryotes. Regulators of m^6^A are involved with tumor proliferation, migration, and invasion among different cancers including gastric cancer, ovarian cancer, bladder cancer, and BRCA (Chen et al., 2019; Niu et al., 2019; Zhang et al., 2019; Liu et al., 2020b; Yi et al., 2020). LncRNAs, the largest class of ncRNAs, mediate their functions including altering cancer progression through interactions with proteins, RNA, DNA, or a combination of these (Qian et al., 2019; Yang et al., 2019). So far, numerous studies have explored the correlations of m^6^A and lncRNA with different cancers. More importantly, new researchers emphasize the role of m^6^A-related lncRNAs in human cancers and tumor microenvironment (Ban et al., 2020; Sun et al., 2020b; Zuo et al., 2020). For instance, m^6^A-related LINC00958 was upregulated in hepatocellular carcinoma cell lines and tissues, the high level of which could independently predict poor OS (Zuo et al., 2020). Overexpression of LNCAROD related to m^6^A methylation took part in malignant development of head and neck squamous cell carcinoma through facilitating YBX1–HSPA1A interaction (Ban et al., 2020). Sun reported that LNC942 targeted METTL14 and regulated the expression and stability of genes CXCR4 and CYP1B1 in BRCA progression. Thus, in this study, a LNC942–METTL14–CXCR4/CYP1B1 signaling axis was put forward (Sun et al., 2020b). These studies indicated the occurrence of m^6^A modulating function commonly in the lncRNAs and potential regulatory mechanism in tumorigenesis. However, the role of m^6^A-related lncRNAs as prognostic biomarkers and their correlations with immune infiltration in BRCA has not been explored. It is essential to investigate the prognostic value of m^6^A-related lncRNAs and their interactions with TIME, a benefit for personalized immunotherapy management.

In this work, to identify sensitive prognostic biomarkers and explore the role in the tumor microenvironment of BRCA, data in TCGA dataset were evaluated by a series of bioinformatics analyses. First, we identified 1,509 lncRNAs associated with 21 m^6^A-related genes from TCGA dataset and RNA-seq data *via* Pearson’s correlation analysis. Besides, 1,089 cases obtained from TCGA were divided randomly into the training cohort and validation cohort. In the two cohorts, the risk scores were calculated based on univariate and multivariate Cox regression analyses and median risk score stratified patients into high- and low-risk groups, respectively. Results revealed that the low-risk group has better OS than the high-risk group. Besides, the prognostic ability of the risk score was further confirmed by the AUC values. Ultimately, through analyzing and comparing the training cohort and validation cohort, 12 m^6^A-related lncRNAs linked closely with prognosis were derived from 1,509 lncRNAs. Additionally, the external validation cohort (196 cases) from our RNA-seq data further confirmed the ability of these signatures in predicting prognosis of BRCA patients. Among these signatures, OTUD6B-AS1 functioned as a prognostic factor for clear cell renal cell carcinoma patients and is mediated through Wnt/β-catenin pathway and the epithelial-to-mesenchymal transition (EMT)-related pathway (Wang et al., 2019). AP002478 served as a prognostic biomarker for patients with *Helicobacter pylori* (+) gastric cancer impacted by three unique pathways (cytokine-cytokine receptor interaction, HIF-1 signaling pathway, and Wnt signaling pathway) (Liu et al., 2019). TGFB2-AS1 induced by transforming growth factor β (TGF-β) was involved in malignant progression of tumors through Smad and protein kinase pathways (Papoutsoglou et al., 2019). These researches exposed the mechanism of lncRNAs in tumors. Furthermore, prognostic signatures based on 12 lncRNAs related with m^6^A were established, which played vital roles in BRCA.

In this study, we explored the roles of tumor microenvironment in BRCA to explain the distinction of survival rates between the high- and low-risk groups. Patients in the low-risk group showed a higher expression of CTLA4, CD274, PDCD1, and CD19, as compared with the high-risk group. The results were consistent with findings of the following studies. Liu et al. (2020a) reported the important roles of CTLA4 and PDCD1 in tumorigenesis, tumor immunity, and prognosis in Pan-Cancer. Park et al. (2020) showed that CD274 expression on tumor cells was associated with prognosis in BRCA patients. Gheybi et al. (2017) disclosed the involvement of CD19 in BRCA’s immune response, linked with outcomes of BRCA patients. Thus, the different expressions of four immune checkpoints were observed, and they may be potential targets for promoting the immunotherapy of BRCA. Similarly, we observed that the low-risk group had higher infiltration levels of 12 immune cells including activated B cell, effector memory CD4 T cell, effector memory CD8 T cell, memory B cell, type 1 T helper cell, type 2 T helper cell, eosinophil, mast cell, natural killer cell, natural killer T cell, and plasmacytoid dendritic cell. Conversely, CD56dim natural killer cell and neutrophil levels were higher in the high-risk group than in the low-risk group. Tekpli et al. (2019) found a new immune-related subtype in BRCA with relevance for prognosis, and the clusters were associated with levels of immune infiltration. The significant survival difference between the two risk subgroups may be related to the distinct expression of immune-oncology targets and immune cell infiltration. Moreover, we estimated the potential effects of SNP on the OS of patients in different groups. Results in the study demonstrated that the difference in the amount of overall gene mutations between the high- and low-risk groups was significant. SNPs are common in the human genome and a universal type of human heritable variation. Many researchers considered SNPs as potential markers in various tumor types, especially BRCA (Gao et al., 2019; He et al., 2019). Due to the effects on cancer risk, analyses of SNPs may help to identify prognostic biomarkers for BRCA therapy.

As revealed in the GSEA results, the tumor functional patterns including Wnt/β-catenin signaling and JAK-STAT signaling pathways were enriched in the high-risk group. In addition, cyclic nucleotide phosphodiesterase activity was identified as the most relevant molecular function of m^6^A-LPS. UV response DN and Wnt/beta-catenin signaling pathway were more associated with the signature. Recent studies have shown that Wnt/β-catenin signaling is involved in BRCA immune microenvironment regulation, proliferation, metastasis, etc. (Xu et al., 2020). For instance, Tang et al. (2019) reported that LncCCAT1 was associated with BRCA progression through Wnt/β-catenin signaling. Considering this, 12 m^6^A-related lincRNAs and these pathways were involved in the differences of BRCA TIME between the two groups. Moreover, results suggested that chemotherapy was beneficial for survival outcome for patients in both the high- and low-risk groups. Through the analysis of the subgroup (patients with chemotherapy), the role of risk score in predicting the chemotherapy response could be noticed. Besides, the expression of ZNF197-AS1 was positively associated with IC_50_ of Nelarabine, ZM-336372, cyclophosphamide, and dexamethasone Decadron.

Twelve m^6^A-related lncRNAs constructed the prognostic signatures for patients with BRCA in TCGA dataset. For further investigation, the risk score could be derived from the prognostic signatures. Univariate and multivariate Cox regression analyses proved that the risk score was an independent prognostic factor for patients with BRCA. Compared with clinicopathological features, risk score displayed more accuracy in predicting prognosis. Furthermore, a nomogram model was established as an applicable quantitative tool to predict the OS of BRCA patients, combining m^6^A-LPS with other clinical features.

It is an undeniable fact that several limitations exist in our study. First, due to the lack of available data about lncRNA sequencing in the Gene Expression Omnibus (GEO) database and other databases, further verification could not be performed. Next, an external verification was performed based on the data from our center, whereas the prognostic follow-up period was insufficient. We will continue to follow up sequencing cases in the future to improve the prediction model. Additionally, the regulation mechanism of m^6^A-related lncRNAs in BRCA TIME is still indistinct and needed further exploration.

In summary, this study constructed an m^6^A-related lncRNA prognostic signature and evaluated the involvement of TIME in BRCA patients. The signature might provide potential targets for accurate prognosis and improvement in immunotherapy for patients with BRCA.

## Materials and Methods

### Datasets

mRNA expression files, sectional lncRNA annotation files, and the corresponding clinical data of BRCA patients were obtained from TCGA data^1^. And the other lncRNA annotation files were derived from RNA-seq data from experiments. Then, we acquired TCGA dataset involving 1,089 patients and RNA-seq data involving 196 BRCA patient samples. Moreover, expression matrixes of 21 m^6^A-related genes included expression data on writers [METTL3, METTL14, METTL16, WTAP, VIRMA (KIA1499), RBM15, RBM15B, and ZC3H13], erasers (FTO and ALKBH5), and readers (YTHDC1, YTHDC2, IGF2BP1, IGF2BP2, IGF2BP3, YTHDF1, YTHDF2, YTHDF3, HNRNPC, HNRNPA2B1, and RBMX), which were obtained from TCGA databases based on previous publications. In addition, we identified 16,501 lncRNAs in TCGA dataset and 17,573 lncRNAs from the RNA-seq data. The profiles for drug response measurements as IC_50_ were downloaded from CellMiner database^2^.

### Bioinformatics Analysis

First, we applied Pearson’s correlation analysis to extract m^6^A-related lncRNAs in each dataset (with the | Pearson R| > 0.5 and *p* < 0.05). The lncRNAs screened from the two datasets were intersected to obtain 1,509 shared lncRNAs. Then, univariate Cox regression analysis and multivariate Cox regression analysis were performed respectively in the training cohort and validation cohort to identify 12 m^6^A-related lncRNAs correlated with the prognoses closely. Thus, an m^6^A-related lncRNA prognostic signature for BRCA patients was developed. The risk score was calculated based on the formula: $R\,i\,s\,k\,s\,c\,o\,r\,e=\sum_{i=1}^{n}C\,o\,e\,f_{i}*X_{i}$. *Coef*_i_ means the coefficients and *X*_i_ means the value of each m^6^A-related lncRNA. Then, we computed the risk scores for all patients including in TCGA dataset. In addition, tumor hallmarks were more common in the high-risk group than the low-risk group by GSEA software. The relative abundance of 28 immune-cell types in the TIME was quantified using single sample GSEA (ssGSEA). For marking immune cell types, special feature gene panels were curated from recent studies (Charoentong et al., 2017; Jia et al., 2018).

### Statistical Analyses

The Kaplan–Meier curves were implemented to compare the different OS between the high- and low-risk groups and other subgroups based on distinct clinicopathological features. Student’s *t*-test was applied to compare the diverse expressions of CTLA4, CD274, PDCD1, and CD19 and numbers of gene mutations in the high- and low-risk groups. Correlation of immune infiltration levels was analyzed by using Pearson’s correlation test. Univariate and multivariate Cox regression analyses were used to assess the independent prognostic value of the m^6^A-LPS. A nomogram was established *via* multivariate Cox regression, and the calibration plots illustrated the accuracy of the nomogram in predicting prognoses. We used ROC curves and the AUC values to evaluate the prognostic abilities of the risk score and other clinicopathological features. The statistical analysis in this study was using R programming language (version 3.6.3), SPSS Statistics 25 software, and GraphPad Prism (version 8.4.0).

## Data Availability Statement

The datasets presented in this study can be found in online repositories. The names of the repository/repositories and accession number(s) can be found in the article/Supplementary Material.

## Author Contributions

XZ, LS, YZ, and XL: conception and design of study. RC: acquisition of the data. XY: analysis and/or interpretation of the data. JY: drafting the manuscript. XW: revising the manuscript critically for important intellectual content. All authors contributed to the article and approved the submitted version.

## Conflict of Interest

The authors declare that the research was conducted in the absence of any commercial or financial relationships that could be construed as a potential conflict of interest.

## Footnotes

1 https://portal.gdc.cancer.gov/

2 https://discover.nci.nih.gov/cellminer/home.do

## Supplementary Material

Supplementary Figure 1 Effector memory CD8 T cell, memory B cell, type 1 T helper cell, type 2 T helper cell, eosinophil, mast cell, natural killer cell, natural killer T cell, plasmacytoid dendritic cell, and CD56dim natural killer cell in two risk groups.

Supplementary Figure 2 **(A)** Kaplan–Meier curves of OS for BRCA patients with high-risk scores based on the chemotherapy. **(B)** Kaplan–Meier curves of OS for BRCA patients with low-risk scores based on the chemotherapy. **(C)** Distribution of risk scores, survival status for BRCA patients in the chemotherapy subgroup. **(D)** ROC curve for patients with 3. 5-, 5-, and 7.5-year survival times in the chemotherapy subgroup.

Supplementary Figure 3 **(A)** Differences of IC_50_ of drugs (nelarabine, ZM-336372, cyclophosphamide, and dexamethasone Decadron) between low and high expression of ZNF197-AS1 groups. **(B)** Correlations of IC50 of drugs (nelarabine, ZM-336372, cyclophosphamide, and dexamethasone Decadron) with various expressions of ZNF197-AS1.

Supplementary Figure 4 Kaplan–Meier curves showed the stratification analysis of the m6A-LPS. The m6A-LPS retained its prognostic value in multiple subgroups of BRCA patients (including patients aged ≤65 or >65 years, female or male, stage I and II or stage III and IV, T1 and 2 or T3 and 4, M0 or ≥M1, N0 or N1–3 and PAM50 molecular subtypes).

Supplementary Figure 5 **(A)** Kaplan–Meier curve of OS for BRCA patients and ROC curve based on the signature including the protein-coding genes. **(B)** ROC curve for the 12 lncRNAs of all BRCA patients in TCGA database.
