# Supplementary figures and images for: Comprehensive Analysis of the Immune-Oncology Targets and Immune Infiltrates of N6-Methyladenosine-Related Long Noncoding RNA Regulators in Breast Cancer
Source: Front Cell Dev Biol. 2021 Jul 2;9:686675. doi: 10.3389/fcell.2021.686675 (PMC8283003; doi:10.3389/fcell.2021.686675)

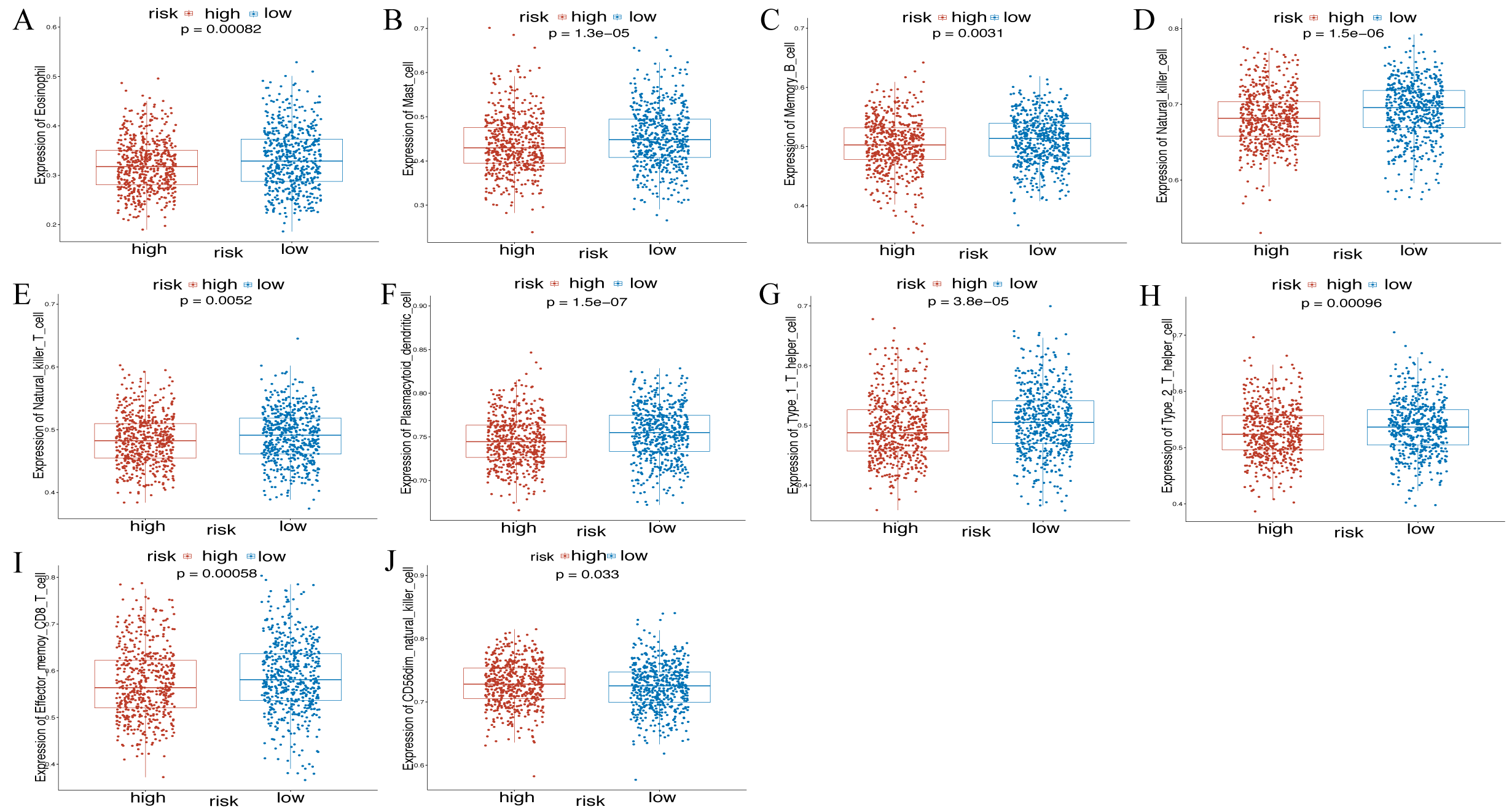

Supplement: Supplementary Figure 1 — Effector memory CD8 T cell, memory B cell, type 1 T helper cell, type 2 T helper cell, eosinophil, mast cell, natural killer cell, natural killer T cell, plasmacytoid dendritic cell, and CD56dim natural killer cell in two risk groups. [file Image_1.TIF]

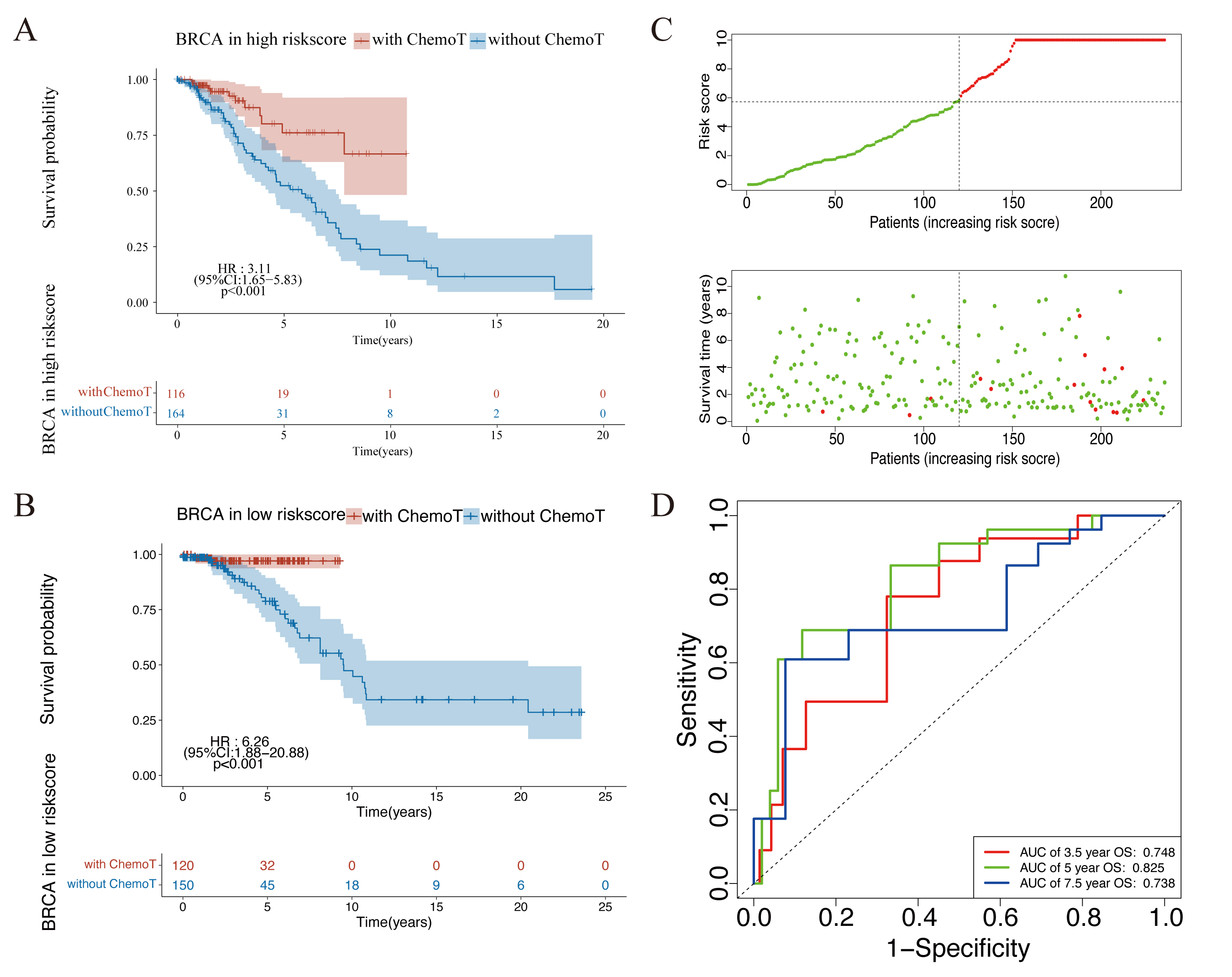

Supplement: Supplementary Figure 2 — (A) Kaplan–Meier curves of OS for BRCA patients with high-risk scores based on the chemotherapy. (B) Kaplan–Meier curves of OS for BRCA patients with low-risk scores based on the chemotherapy. (C) Distribution of risk scores, survival status for BRCA patients in the chemotherapy subgroup. (D) ROC curve for patients with 3. 5-, 5-, and 7.5-year survival times in the chemotherapy subgroup. [file Image_2.TIF]

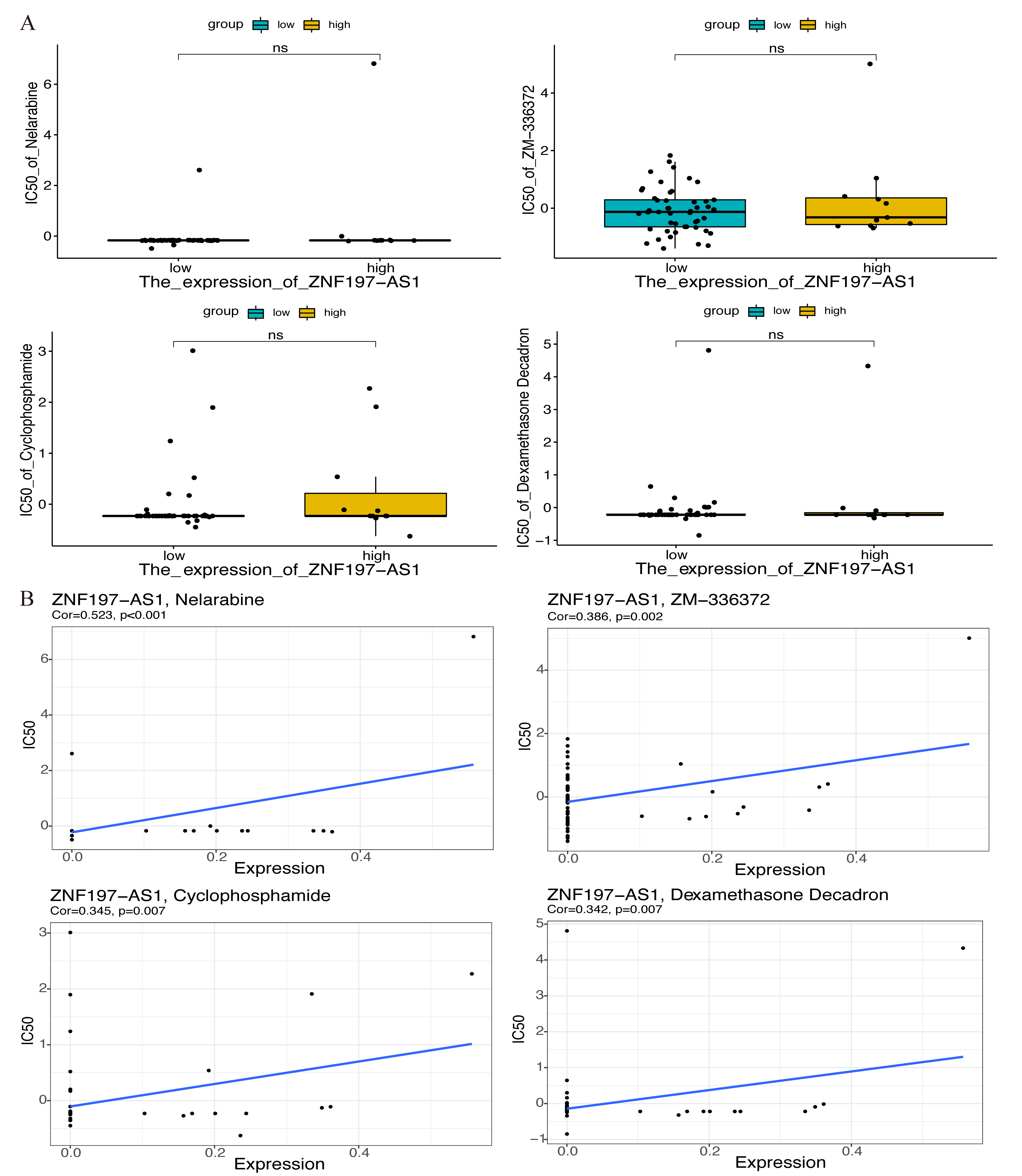

Supplement: Supplementary Figure 3 — (A) Differences of IC50 of drugs (nelarabine, ZM-336372, cyclophosphamide, and dexamethasone Decadron) between low and high expression of ZNF197-AS1 groups. (B) Correlations of IC50 of drugs (nelarabine, ZM-336372, cyclophosphamide, and dexamethasone Decadron) with various expressions of ZNF197-AS1. [file Image_3.TIF]

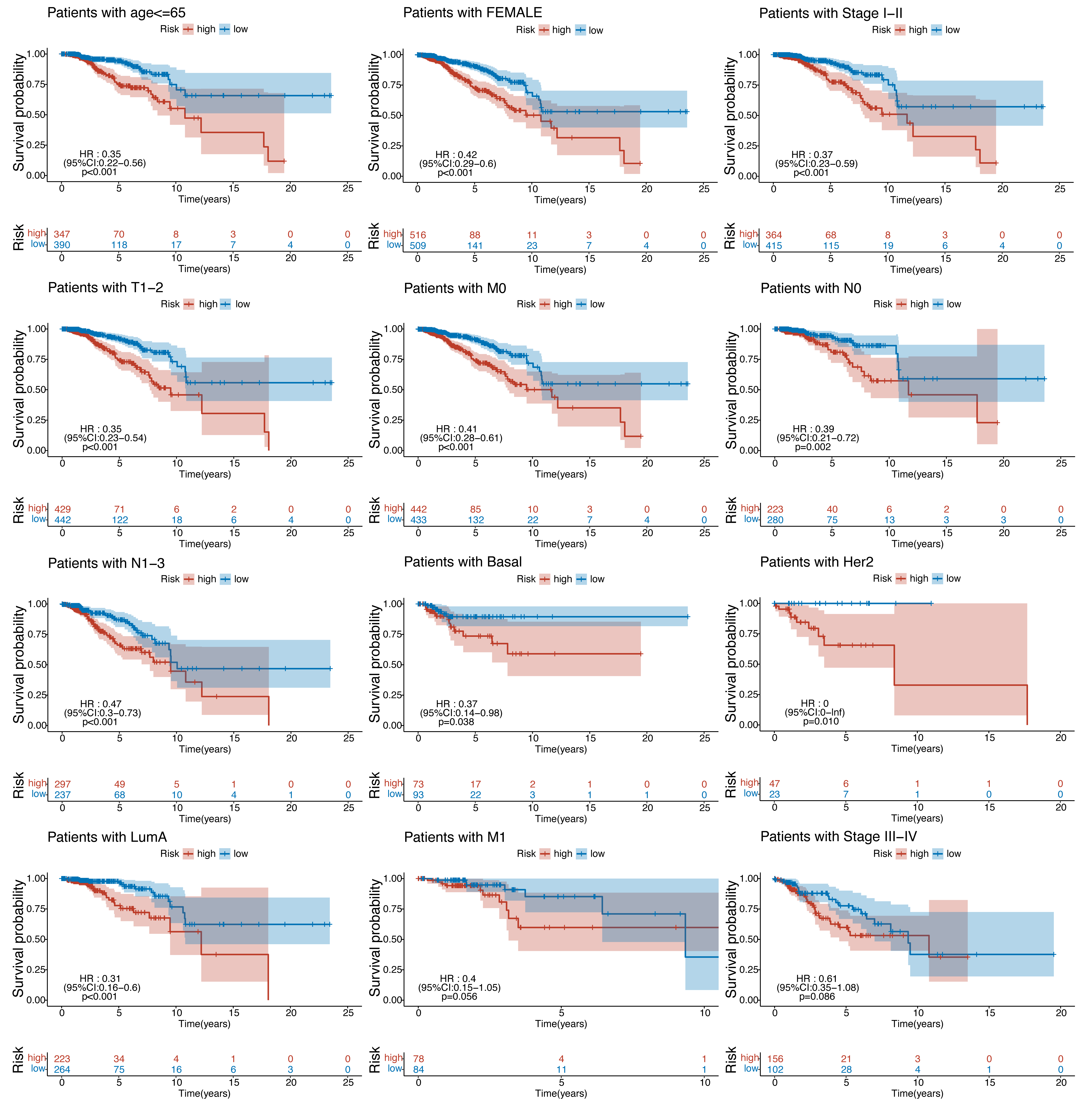

Supplement: Supplementary Figure 4 — Kaplan–Meier curves showed the stratification analysis of the m6A-LPS. The m6A-LPS retained its prognostic value in multiple subgroups of BRCA patients (including patients aged ≤65 or >65 years, female or male, stage I and II or stage III and IV, T1 and 2 or T3 and 4, M0 or ≥M1, N0 or N1–3 and PAM50 molecular subtypes). [file Image_4.TIF]

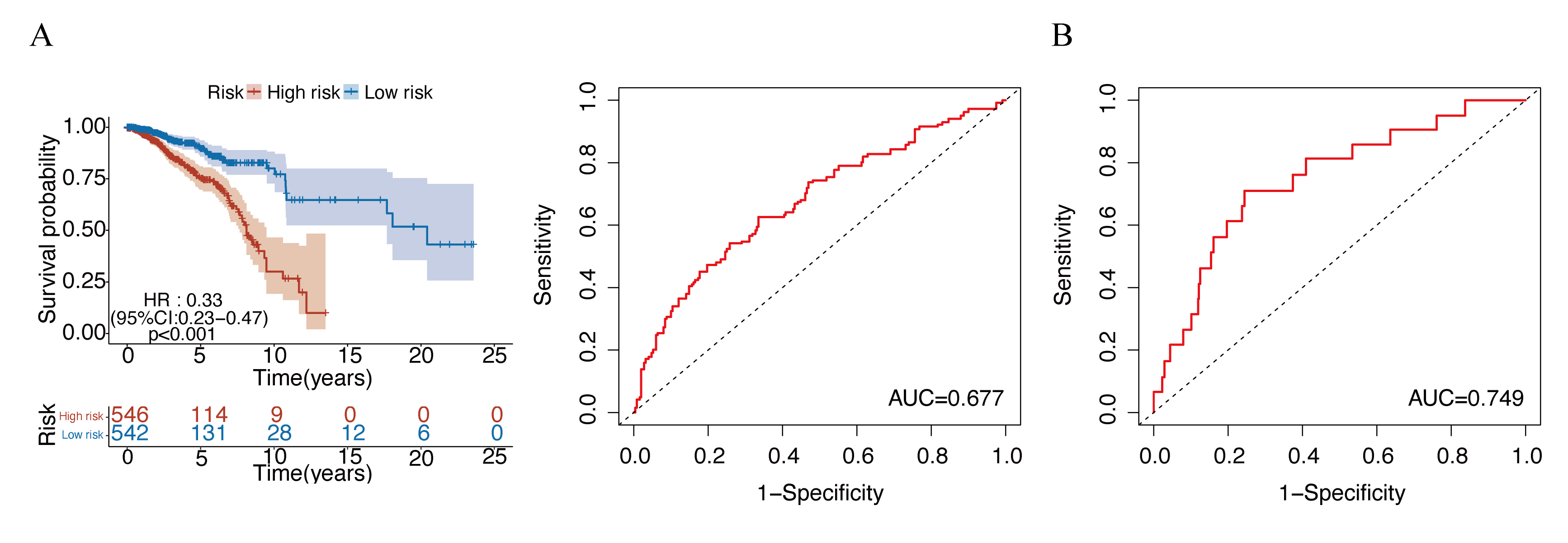

Supplement: Supplementary Figure 5 — (A) Kaplan–Meier curve of OS for BRCA patients and ROC curve based on the signature including the protein-coding genes. (B) ROC curve for the 12 lncRNAs of all BRCA patients in TCGA database. [file Image_5.TIF]
